# Giant Right Coronary Artery Aneurysm Fistulization to the Coronary Sinus: Technique for Surgical Repair

**DOI:** 10.1016/j.atssr.2026.02.015

**Published:** 2026-02-27

**Authors:** Matthan Tharakan, Mathew K. George, Mathew J. Tharakan, Emmanuel Li, Holden Husbands, Ajit K. Tharakan

**Affiliations:** 1 University of Oklahoma Health Sciences Center, Oklahoma City, Oklahoma; 2 Texas Tech University Health Science Center, Lubbock, Texas; 3 Saveetha Medical College, Chennai, India; 4 Oklahoma Heart Institute, Tulsa, Oklahoma

## Abstract

Fistulas from the right coronary artery (RCA) to the coronary sinus (CS) are rare coronary anomalies, and their coexistence with diffuse RCA aneurysmal disease is even less common. We report a 76-year-old woman with severe symptomatic mitral regurgitation found to have a large, tortuous aneurysmal RCA draining through a large fistula into the CS. In patients with diffusely aneurysmal RCA anatomy, intracardiac closure from within the CS can avoid extensive epicardial dissection while preserving coronary venous drainage. Complex coronary anomalies should not preclude definitive valve and rhythm surgery when myocardial protection strategies are tailored to the underlying anatomy.

Coronary artery fistulas (CAFs) are rare anomalies characterized by a communication between a coronary artery and a cardiac chamber or great vessel.^1^ Although CAFs most commonly drain into right-sided chambers or the pulmonary artery, fistulous connections from the right coronary artery (RCA) to the coronary sinus (CS) are particularly uncommon and have been described only in isolated case reports. Coronary artery aneurysms, defined as focal dilatations ≥1.5 times the diameter of an adjacent normal segment, occur in 0.3% to 5% of patients undergoing coronary angiography and may coexist with CAFs, further increasing the risk of thrombosis, rupture, or myocardial ischemia.^2^ Surgery is favored for large, tortuous, or multicomponent lesions and when concomitant cardiac procedures are required.^3^ We present a case of severe degenerative mitral regurgitation with pulmonary hypertension in the setting of a large aneurysmal RCA-to-CS fistula, highlighting operative strategies to achieve durable correction of valvular and coronary anomalies.

A 76-year-old woman with a history of hypertension, hyperlipidemia, pulmonary hypertension, and atrial fibrillation was referred for evaluation of severe symptomatic degenerative mitral regurgitation. She reported progressive exertional dyspnea and orthopnea over 3-6 months. Transthoracic echocardiography demonstrated a normal-sized left ventricle with mild concentric hypertrophy and preserved systolic function (ejection fraction, 0.64). There was biatrial enlargement and myxomatous degeneration of the mitral valve with profound prolapse of the P2 segment of the posterior leaflet resulting in severe (4+) mitral regurgitation. Additional findings included moderate (2+) tricuspid regurgitation and elevated pulmonary artery systolic pressure estimated at 69 mm Hg.

Cardiac computed tomography showed an enlarged left ventricle with normal systolic function and an enlarged right ventricle with mildly reduced systolic function (Figure 1). Cardiac catheterization revealed angiographically mild-to-moderate coronary artery disease with a large tortuous RCA aneurysm with a fistulous connection into the CS (Figure 2).

**Figure 1:**
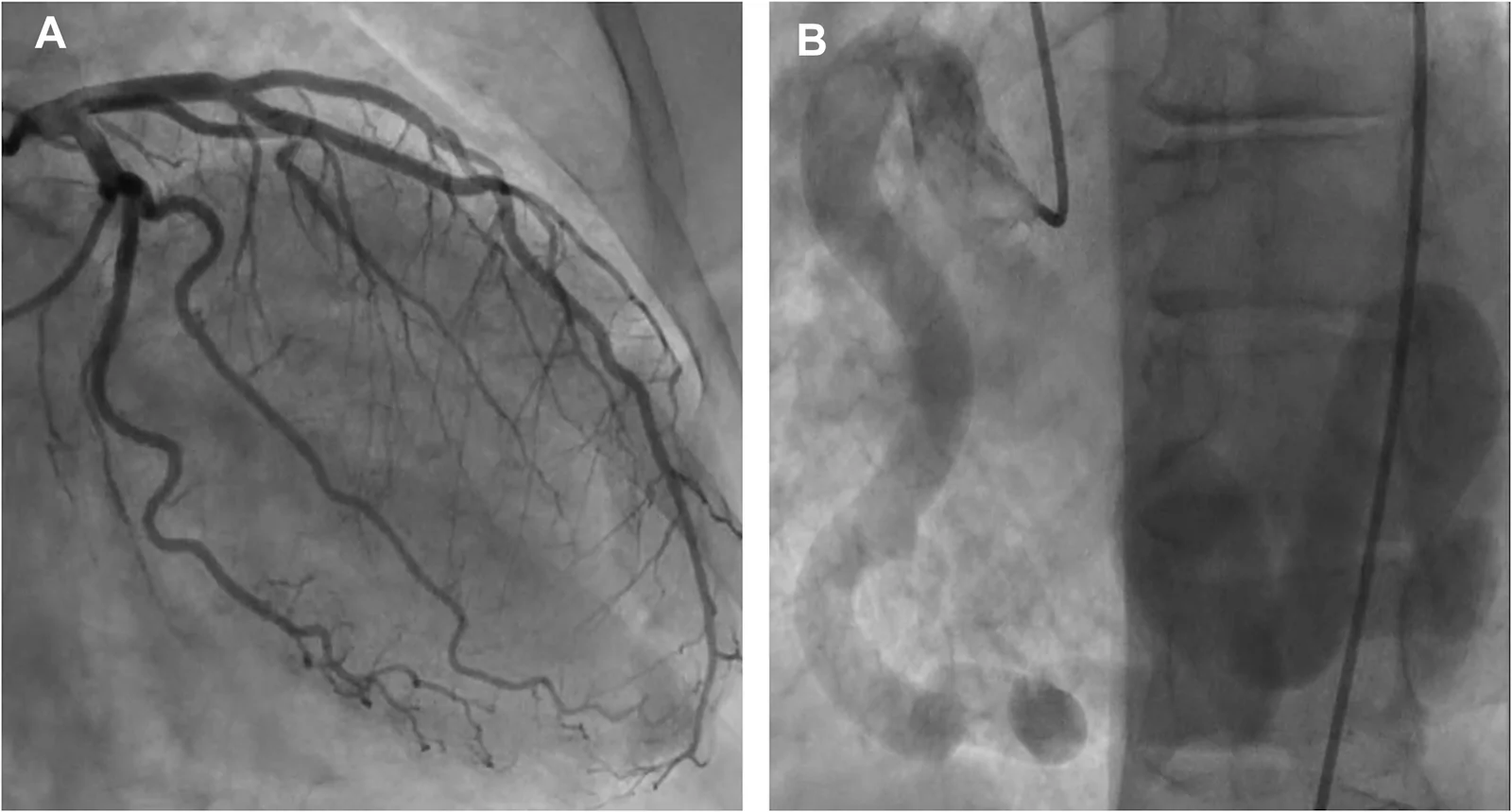
(A) A left coronary angiogram demonstrates right coronary artery–to–coronary sinus fistula. (B) The large aneurysmal segment is shown coursing inferiorly and draining into an enlarged coronary sinus.

**Figure 2:**
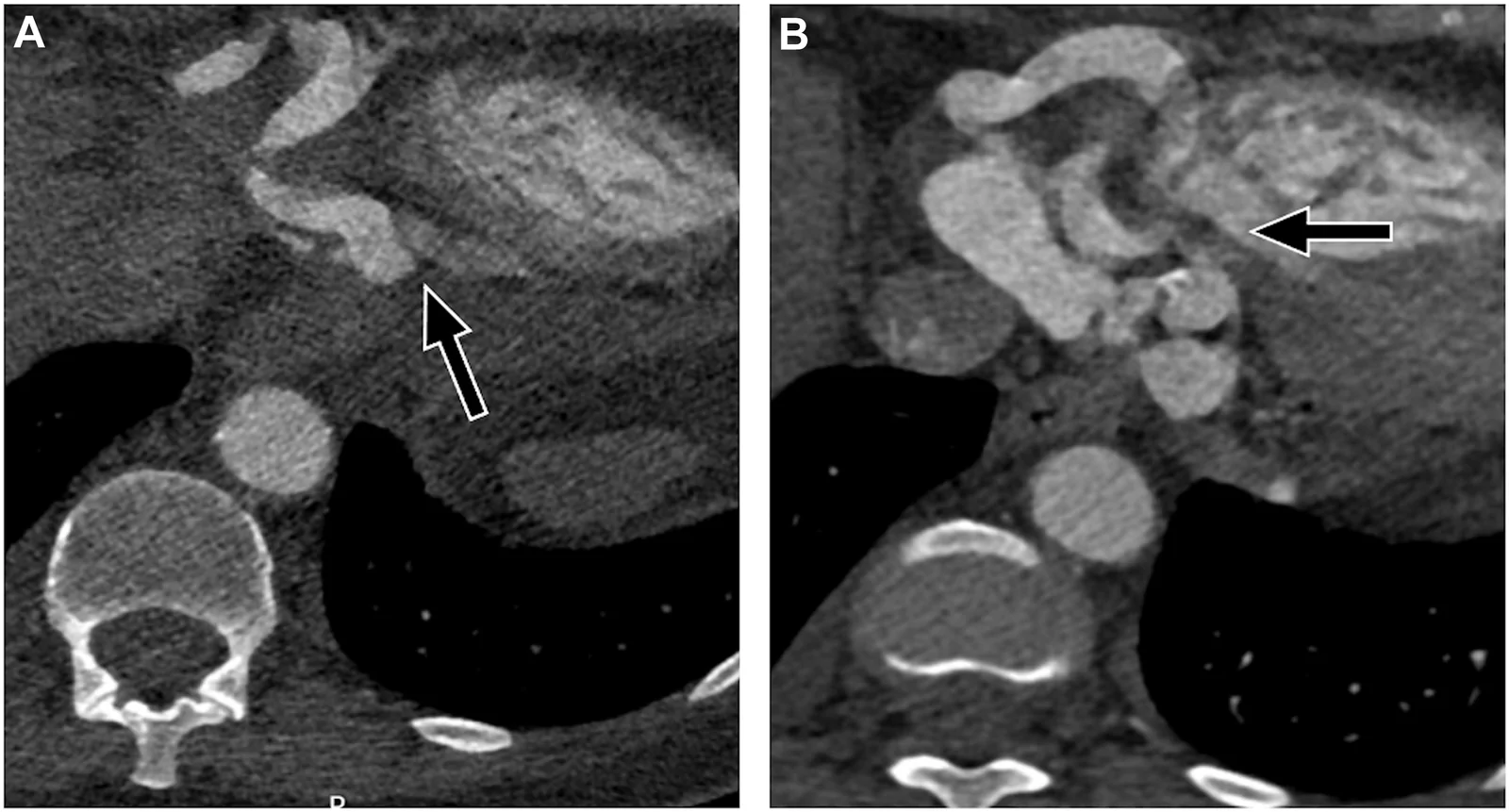
Cardiac computed tomography (CT) demonstrates right coronary artery aneurysm with fistulous drainage into the coronary sinus. (A) Axial contrast-enhanced CT image shows a right coronary artery coursing along the right atrioventricular groove indicated by the arrow. (B) Axial slice illustrates the contrast-filled right coronary artery aneurysm as it communicates with an enlarged coronary sinus (arrow).

After median sternotomy, the large aneurysm of the RCA was immediately visible (Figures 3A, 3B). The patient was fully heparinized, and cardiopulmonary bypass was established using an ascending aortic cannulation and bicaval venous cannulation. The aorta was cross-clamped, and antegrade blood cardioplegia was administered through the aortic root; however, electromechanical arrest could not be achieved despite multiple doses of antegrade cardioplegia. This was due to runoff through the RCA-to-CS fistula.

**Figure 3:**
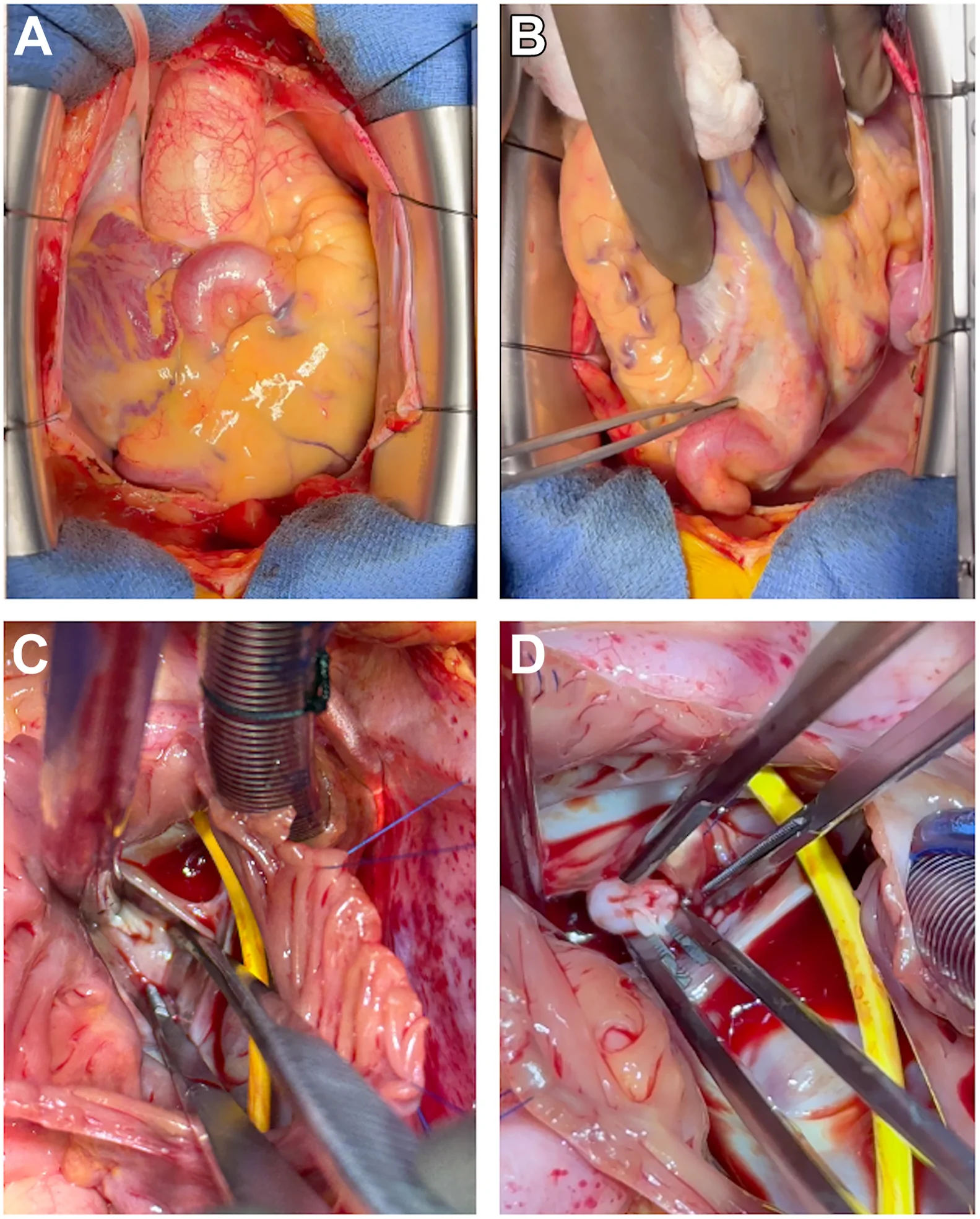
Intraoperative findings and repair of a right coronary artery aneurysm with fistulous drainage to the coronary sinus. (A, B) Initial median sternotomy exposure of the aneurysmal right coronary artery. (C) Operative field with exposure of the coronary sinus and identification of the fistulous orifice. (D) Direct closure of the right coronary artery–to–coronary sinus fistula from within the right atrium.

Given inadequate myocardial arrest, attention was first directed to ligating the fistula. The right atrium was opened parallel to the atrioventricular groove, providing excellent exposure. The CS was unroofed, and the incision was extended through the valve of the CS into the main body of the terminal CS. The fistulous opening of the fistula into the CS was identified by giving short doses of antegrade blood cardioplegia to definitively determine the opening (Figures 3C, 3D; Supplemental Video 1). The fistula was then ligated using pledgeted 5-0 Prolene (Ethicon) sutures. Repeat doses of antegrade cardioplegia confirmed the absence of further leakage through the fistula.

The right atrium was closed in 2 layers. After this repair, subsequent doses of antegrade cardioplegia produced prompt and complete electromechanical arrest of the heart. The left atrium was opened through an inferior approach for good exposure of the mitral valve. Inspection revealed a flail P2 segment of the posterior leaflet. The mitral repair was completed using a quadrangular resection of P2 and ring annuloplasty was completed using a 28-mm Physio 2 ring (Edwards Lifesciences), with good result as confirmed after saline testing and pressurizing the left ventricle. A transesophageal echocardiogram after the repair demonstrated a well-functioning reconstructed mitral valve, with only trace residual regurgitation and a mean transmitral gradient of 2 mm Hg. Biventricular function was good, with no residual shunt from the RCA into the CS or right atrium.

The patient made an uneventful postoperative recovery and was discharged on postoperative day 4. The patient was seen for follow-up after 6 weeks. Patient was doing well, with no postoperative complications.

## Comment

Several case reports have described RCA aneurysm with CS fistula, but with different anatomic patterns and operative strategies. Özler and colleagues^4^ reported an RCA aneurysm with a fistulous communication to the CS treated by complete resection of the aneurysm and distal division of the fistula at its CS insertion, showing a previous surgical approach aimed at eliminating both the shunt and the aneurysmal segment. In contrast, Briosa e Gala and colleagues^5^ described a giant proximal RCA aneurysm with a CS fistulation with a computed tomography angiography showing a large saccular aneurysm and right-sided volume overload. The patient underwent RCA ostial repair with a pericardial patch and aneurysm decompression with an excellent early outcome.^5^

Compared with these cases, our patient had a diffusely aneurysmal RCA, with substantial left-to-right shunt into a dilated CS, and a concurrent degenerative mitral valve. Management options for coronary artery aneurysm with fistula now range from conservative medical therapy to surgical intervention.^6^ Our case adds to this spectrum by emphasizing intracardiac control of the fistula from within the CS to restore a closed coronary circuit and allow myocardial protection, while avoiding extensive dissection of the RCA aneurysm.

Our decision to close the fistula first was due to cardioplegia failing initially. Immediate fistula repair allowed us to repair the mitral valve repair safely. Preoperative imaging should anticipate the impact of the shunt on cardioplegia strategy, and surgeons should be prepared to prioritize early fistula repair if cardioplegia runoff is encountered.

Our decision to leave the RCA after secure elimination of the CS shunt reflects an area where data are sparse. Existing observational studies and reviews of coronary artery aneurysms, including those with associated fistulas, suggest that long-term outcomes are influenced by aneurysm size and the extent of shunt or aneurysm elimination.^7^ However, they do not provide clear guidance on when a residual nonstenotic segment should be surgically removed vs managed with antithrombotic therapy.^7^ Likewise, there is no consensus in the general literature on optimal antithrombotic regimens after combined fistula repair and valve surgery. Future multicenter registries focusing specifically on coronary-to-CS fistulas and aneurysmal variants are needed to refine indications for transcatheter vs surgical repair and clarify when aneurysm resection is necessary.
